# Improving a training model for vascular surgical techniques

**DOI:** 10.1590/1677-5449.190032

**Published:** 2019-09-24

**Authors:** Adenauer Marinho de Oliveira Góes, Edson Yuzur Yasojima, Rosa Helena de Figueiredo Chaves, Flávia Beatriz Araújo de Albuquerque

**Affiliations:** 1 Universidade Federal do Pará – UFPA, Belém, PA, Brasil.; 2 Centro Universitário do Estado do Pará – CESUPA, Belém, PA, Brasil.

**Keywords:** surgery, vascular surgical procedures, anastomosis, models, medical education

## Abstract

We describe a low-cost model for training vascular surgical techniques. The model is constructed from cylindrical latex balloons filled with gelatin and fixed to a board for support. Arterial sutures, end-to-side and end-to-end anastomoses, patch, vascular shunt placement, and thromboembolectomy were simulated.

## INTRODUCTION

Construction of vascular anastomoses is a common procedure in many types of surgery. This is because its primary objective is reestablishment of blood flow to organs and tissues, which is a procedure that is necessary in many different specialties in addition to vascular surgery itself, including trauma surgery, organ transplantation, and construction of patches and reimplantation in plastic surgery.^1^^-^ 4

Currently, the majority of general surgery residents’ training is conducted on human beings, with demonstrations by more experienced surgeons.^1^^,^ 5 However, construction of vascular anastomoses requires significant exposure, dissection, and temporary occlusion of the vessel, increasing the risk of complications, particularly when performed by an inexperienced surgeon.^6^ The best way to acquire and develop this skill is through training with experimental models.^4^

Simulators are inanimate models developed for training a specific technical or motor skill; and simulations are the various different situations in which use of this technical skill is part of the competence being trained.^2^ Simulations are important in medical education because they enable a range of different skills to be trained, improving patient safety.^5^^,^ 7

Another objective of simulation is to develop simpler and more functional training methods, using the lowest possible number of animals for experimentation, thereby adhering to the 3 Rs policy (refinement, replacement, and reduction).^4^ This is why the American College of Surgeons considers use of skills training models and simulators for accreditation of specialists rather than animal models.^8^

Among the training models most widely used today, “bench models” are of particular interest because they employ inanimate materials – whether artificial (rubber or foam structures) or biological (bovine tongue and other animal viscera) – that are low cost, but enable the basic principles of surgery to be taught.^3^^,^ 4

There are many different biological models for practicing vascular anastomoses, mostly involving animal viscera.^4^^-^ 7 The current initiative within the scientific community is to diversify use of already-existing teaching models to continue the trend to reduce use of animals, in addition to reducing risks and optimize surgery time in humans.^4^^,^ 5

## OBJECTIVE

To present a reproducible, low-cost experimental model for training vascular anastomoses that can also be adapted for other surgical techniques, such as patches and embolectomy, using inflatable balloons and gelatin.

## METHOD

This is an experimental study describing an application of inflatable balloons and gelatin to construct a model for training vascular surgical techniques.

Materials employed to construct the model and perform the procedures:

Latex balloons (28 cm long and 5 mm in diameter) colored red, blue, and white to simulate arteries, veins, and synthetic tissue, respectively; a white plastic kitchen chopping board (size: 40.5 cm x 26.0 cm x 7.0 mm); a 110 g pot of commercial gelatin (of the type sold for children to play with); double-sided adhesive tape; 5.0 polypropylene cardiovascular surgery sutures; a 5 mL syringe; a number 4 Fogarty catheter®; and a vascular shunt (vascushunt – Edwards Lifesciences®). Instruments used: Mayo Hegar tungsten carbide-tipped needle holders; two Bulldog Dieffenbach clamps; two curved and two straight Kelly clamps; a Debakey tweezer and Mayo scissors.

Using eight latex balloons, it was possible to simulate the surgical techniques described below.

### Assembly and use of the model:

**Figure 1:**
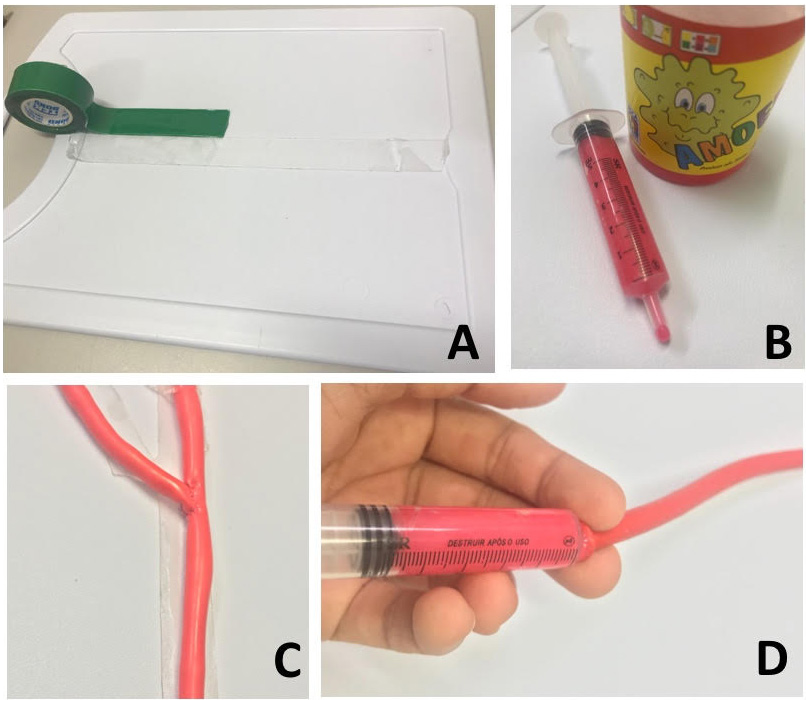
Materials employed to assemble the model: plastic board and double-sided adhesive tape (A); 5 mL syringe and gelatin (B); model of the carotid bifurcation already fixed to the plastic board (C); injection of gelatin into the balloon using the 5 mL syringe (D).

The 5 mL syringe was used to inject 10 mL of gelatin into the lumen of each balloon, tying a knot in the open extremity to maintain the contents inside. External compression maneuvers were used to distribute the gelatin content uniformly along the length of the balloon. The balloon was then attached to the plastic board using double-sided tape (Figure 1).

**Figure 2:**
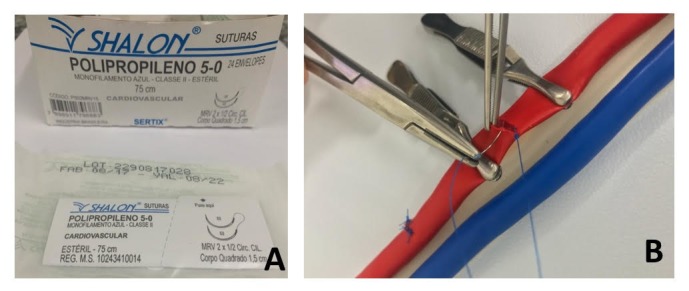
Vascular sutures: threads used (A); simulation of arteriorrhaphy (B).

Grafts and patches were constructed according to the basic principles of vascular anastomosis described by Carrel,^9^ Guthrie,^10^ and Rutherford,^11^ using 5.0 polypropylene cardiovascular sutures (Figure 2).

**Figure 3:**
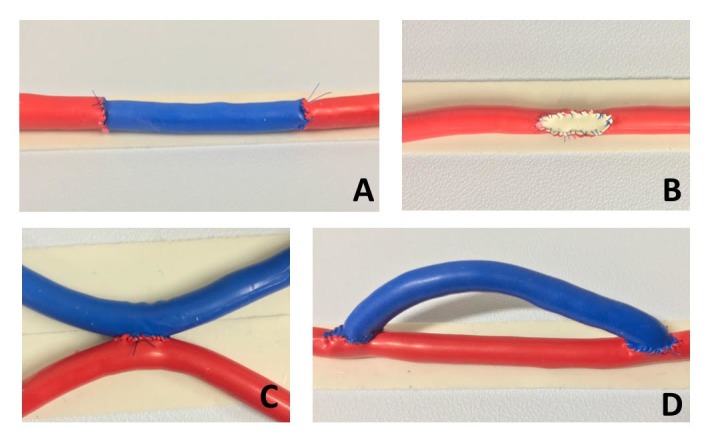
Simulations performed: graft with end-to-end anastomoses (A); patch (B); side-to-side anastomosis (C); and graft with end-to-side anastomoses (D).

**Figure 4:**
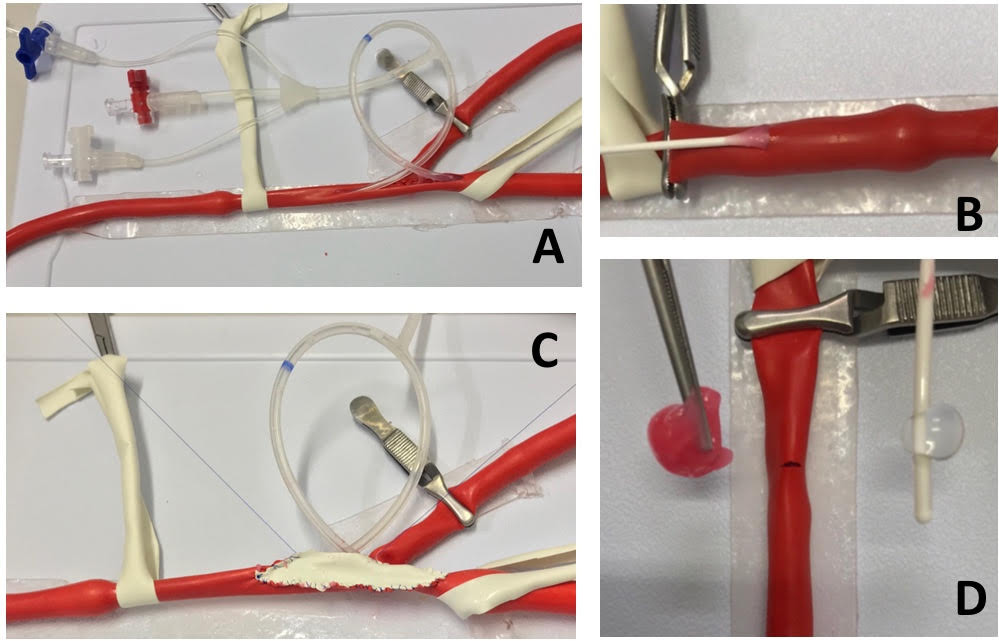
Carotid endarterectomy scenario: implant of carotid shunt (A) and construction of patch (C). Simulation of thromboembolectomy: traction of inflated catheter in an intraluminal position (B); fragment of gelatin removed (simulating the thrombus) and the Fogarty catheter® used (D).

Several different types of anastomoses were tested using the same experimental model: end-to-end, side-to-side, and end-to-side, as shown in Figure 3. Vascular shunt insertion and patching were also tested, simulating a carotid endarterectomy, and a thromboembolectomy was simulated using a Fogarty catheter® (Figure 4).

Patency of the anastomoses was confirmed by redistribution of the intraluminal gelatin after removal of the Bulldog clamps and leakage of gelatin between the stitches of vascular sutures was also evaluated (anastomoses, lateral sutures, and patch).

## RESULTS

After completion of the sutures, good coaptation of edges was observed and all anastomoses were patent, as demonstrated by redistribution of the intraluminal gelatin after release of the clamps.

It proved possible to perform the following vascular surgical techniques: lateral suture (arteriorrhaphy), end-to-end, side-to-side, and end-to-side anastomoses, simulating construction of grafts and patches, thromboembolectomy, and placement of a temporary vascular shunt.

No significant leakage of gelatin was seen, but, since this is a colloid substance, with gradual dispersal, discrete leakage between stitches was observed and in some cases through the orifices created by transfixing the balloon with the needle.

The total cost of the model produced was R$ 88.99, as illustrated in Table 1. This does not include the costs of permanent materials, such as the surgical instruments.

**Table 1:** Costs related to the training model.

**Description**	**Unit cost**
Bag of cylindrical balloons containing 50 units	4.49
Plastic chopping board	5.59
Double-sided adhesive tape	10.50
Box of sutures (5.0 polypropylene cardiovascular sutures) containing 24 units	68.41
Total	88.99

## DISCUSSION

Use of simulators to hone surgical skills was validated many years ago; but cadavers and experimental animals are still the models most widely used.^4^ Since the Arouca Law (nº 11.794/08)^12^ was passed, one of the scientific community’s objectives has been to diversify simulation models, reducing use of animals and enabling repetitive training of surgical techniques, producing better-prepared newly-qualified surgeons and reducing patient risk.

Many different models for training vascular sutures have been described in the literature. Using surgical microscopes, both Webster and Ely^13^ and Lima et al.^14^ have described training for sutures and end-to-end anastomoses using 0.6 and 1 mm silicone tubes and boards. They used nylon threads with gauges varying from 8-0 to 10-0 and after initial training with silicone, used live rats and the limbs of slaughtered birds to refine the technique.^13^^,^ 14

Models using organic materials include chicken trachea and esophagus, simulating the consistency of arteries and veins respectively,^7^ vessels from bovine tongue,^4^ and recently-amputated human limbs.^5^ However, precautions must always be taken when using these materials because of the risk of biological contamination. These precautions include: use of protective devices such as gloves, masks, and goggles, appropriate disposal of the organic material, and care with laboratory hygiene.

A model described by Grahem et al.^1^ employed vegetables with tubular structures, such as green beans and yardlong beans, describing it as a low-cost model for training end-to-end anastomosis. The consistency and malleability of those vegetables probably would not be suitable for more refined techniques, such as the end-to-side anastomoses, patches, and others described in the present study.

Use of latex balloons for training vascular anastomoses was described by Sarmento et al.,^15^ who reported that their malleability, cylindrical shape, thinness, and internal lumen were all similar to blood vessels. In that model, the balloons were not filled with any intraluminal contents, in contrast with the present study, in which gelatin was used, so that the balloons would remain turgid. Another difference in relation to the model previously proposed is the balloons’ attachment: in the previous model, the balloons were suspended by screws over a supporting board and were not maintained in contact with its surface.

In a pilot phase of this study, the balloons were fixed to the boards at their extremities only, using metal staples. However, it was found that when the balloon was fully sectioned transversely, prior to end-to-end anastomosis, it became loose, being attached only by the staples. In a real-life situation, vessels remain attached to the adjacent tissues, conferring relative immobility and facilitating sutures, which is why the staples were substituted with double-sided tape along the entire length of the balloon.

Without increasing the cost unduly, the adherence provided by the tape and maintenance of the balloon’s turgidity with the gelatin made the model more faithful. These modifications also made it possible to train not only sutures and anastomoses, but also more complex procedures, such as thromboembolectomy and placement of vascular shunts.

In addition to the low cost, other advantages observed include the fact that along the length of a single balloon (28 cm), several vascular sutures/anastomoses can be practiced and, since none of the materials employed are perishable, they can be stored for long periods.

In the majority of low-cost models of vascular anastomosis, one limitation is related to evaluating the quality of the distance between stitches, since in these synthetic models there are no coagulation factors to reduce leakage between stitches.^1^^,^ 15 Even though spacing of approximately 1 mm between stitches was maintained, discrete leakage of gelatin was observed, which was expected because of the physical characteristics of the material. However, the peculiar expansivity of the gelatin also made it possible to attest to the patency of anastomoses, since once the clamps had been removed, the gelatin moves to fill the space; other models depend on intraluminal injection of liquid to test the patency of anastomoses.^1^^,^ 5 ^,^ 7 ^,^ 15

During the pilot phase, a model was tested using 6-0 polypropylene sutures. Although it was possible to complete all of the procedures, the suture must be tractioned more carefully, because the friction between the thread and the latex of the balloon can cause the suture to break more easily when pulled. Numbers 3 and 4 Fogarty catheters were also tested. While it was possible to remove intraluminal gelatin with both of them, it was found during the initial phase of training that with the number 3 catheter the balloon may burst more easily during manipulation.

We consider that it is worthwhile setting a training schedule that correlates all of the different techniques simulated, with the objective of representing what would be encountered in real situations. For example: for “construction” of an arterial bifurcation, it is necessary to perform an end-to-side anastomosis, followed by (on the same structure) a transverse “arteriotomy” simulating an embolectomy at the level of the femoral bifurcation, or a longitudinal “arteriotomy” (as at the carotid sinus), demonstrating placement of a shunt and synthesis with a patch.

This model was initially developed for a trauma surgery course developed by one of the authors and delivered at our institution. On that course, general surgery residents simulated arteriorrhaphy and end-to-end anastomoses. The model was later refined for use to train the other procedures described. During this phase, simulations of procedures were performed by one of the authors, a vascular surgeon, aided by undergraduate research fellows attached to the experimental research team at our Medical Faculty. Our objective in publishing this article was to share the instructions for assembly of the model; the study will be continued and will assess the degree of satisfaction and the impact on users’ training.

The procedures were conducted on this model with and without the aid of optical magnification with a microsurgery loupe and we believe that the model can also be used for technical refinement, to practice use of optical magnification.
