# Supplementary material for: Improving a training model for vascular surgical techniques
Source: J Vasc Bras. 2019 Sep 24;18:e20190032. doi: 10.1590/1677-5449.190032 (PMC6774658; doi:10.1590/1677-5449.190032)
Supplement: Translation

# Translation: Aperfeiçoando um modelo de treinamento para técnicas cirúrgicas vasculares

**Authors:** Adenauer Marinho de Oliveira Góes, Edson Yuzur Yasojima, Rosa Helena de Figueiredo Chaves, Flávia Beatriz Araújo de Albuquerque

**Affiliations:** 1 Universidade Federal do Pará – UFPA, Belém, PA, Brasil.; 2 Centro Universitário do Estado do Pará – CESUPA, Belém, PA, Brasil.

**Keywords:** cirurgia, procedimentos cirúrgicos vasculares, anastomose, treinamento, educação médica

## Abstract

Descreve-se um modelo de baixo custo para o treinamento de técnicas cirúrgicas vasculares; o modelo foi montado com balões cilíndricos de látex, preenchidos com gelatina e fixados a uma placa de suporte. Foram simuladas arteriorrafias, anastomoses término-laterais e término-terminais, *patch*, colocação de *shunt* vascular e tromboembolectomia.

## INTRODUÇÃO

A elaboração de anastomoses vasculares é uma conduta comum em diversas cirurgias. Isso ocorre porque seu principal objetivo é reestabelecer o fluxo sanguíneo para órgãos e tecidos, procedimento necessário em várias especialidades além da própria cirurgia vascular, como a cirurgia do trauma, transplantes de órgãos e realização de retalhos e reimplantes por cirurgiões plásticos^1^^-^ 4.

Atualmente, a maioria dos treinamentos de residentes em cirurgia geral é realizada em seres humanos, por meio da demonstração por cirurgiões mais experientes^1^^,^ 5. Contudo, a confecção de anastomose vascular requer extensa exposição, dissecção e oclusão temporária do vaso, aumentando o risco de complicações, principalmente se realizada por cirurgião inexperiente^6^. A melhor forma de adquirir o desenvolvimento dessa habilidade é o treinamento em modelos experimentais^4^.

Simuladores são os modelos inanimados desenvolvidos para o treinamento de determinada prática técnica/motora; e simulação são as diferentes situações em que o emprego da habilidade técnica é parte da competência a ser desenvolvida^2^. A simulação é importante no ensino da medicina por permitir o treinamento de diversas habilidades, proporcionando segurança ao paciente^5^^,^ 7.

A simulação também tem o objetivo de desenvolver métodos mais simples e funcionais de treinamento, utilizando o mínimo possível de animais em experimentação, cumprindo os princípios dos 3 Rs (*refinement, replacement e reduction*)^4^. Por esse motivo, o Colégio Americano de Cirurgiões considera a utilização de modelos de treinamento em habilidades e de simuladores para acreditação de especialistas em detrimento dos modelos animais^8^.

Entre os modelos de ensino mais usados hoje, os chamados “modelos de bancada” são de interesse por utilizarem materiais inanimados – artificiais (estruturas de borracha ou espuma) ou biológicos (línguas bovinas e outras vísceras animais) – de baixo custo, mas que permitem o ensino de princípios cirúrgicos básicos^3^^,^ 4.

Existem diversos modelos biológicos para a prática de anastomoses vasculares, principalmente envolvendo vísceras animais^4^^-^ 7. A iniciativa atual da comunidade científica é diversificar a utilização de modelos de ensino já existentes para continuar a tendência na redução da utilização de animais, além de diminuir riscos e otimizar o tempo de cirurgia em humanos^4^^,^ 5.

## OBJETIVO

Apresentar um modelo experimental reproduzível e de baixo custo para o treinamento de anastomoses vasculares e que também seja adaptável para outras técnicas cirúrgicas, como remendos (*patch*) e embolectomia, utilizando balão inflável e gelatina.

## MÉTODO

Trata-se de estudo experimental descrevendo a aplicação de bexiga inflável e gelatina para a confecção de um modelo de treinamento de técnicas cirúrgicas vasculares.

Materiais utilizados para confecção do modelo e realização dos procedimentos:

Foram utilizados balões de látex (28 cm de comprimento e 5 mm de diâmetro) nas cores vermelho, azul e branco, simulando artéria, veia e tecido sintético respectivamente; uma “tábua” de cozinha feita de plástico na cor branca (tamanho 40,5 cm x 26,0 cm x 7,0 mm); um pote de 110 g de gelatina industrial (do tipo destinado à utilização recreativa infantil); fita adesiva dupla face; fio cirúrgico de polipropileno cardiovascular 5.0; uma seringa de 5 mL; um cateter de Fogarty® número 4; e um *shunt* vascular (*vascushunt* – Edwards Lifesciences®). Instrumentais utilizados: um porta-agulha Mayo Hegar com ponta de vídia; duas pinças Bulldog Dieffenbach; duas pinças Kelly curvas e duas retas; uma pinça Debakey; e uma tesoura de Mayo.

Utilizando oito balões de látex, foi possível fazer a simulação das técnicas cirúrgicas descritas a seguir.

### Montagem e utilização do modelo

Com uma seringa de 5 mL, foram injetados 10 mL de gelatina no lúmen de cada balão, dando-se um nó na extremidade aberta para manter o conteúdo no seu interior, o qual, com o auxílio de manobras de compressão externa, foi homogeneamente distribuído ao longo da extensão do balão. Posteriormente, o balão foi fixado à placa de plástico por meio da fita adesiva dupla-face (Figura 1).

**Figura 1:** Materiais utilizados na confecção do modelo: “tábua” de plástico e fita adesiva dupla face (A); seringa de 5 mL e gelatina (B); modelo de bifurcação carotídea já fixado à “tábua” plástica (C); introdução da gelatina no balão utilizando a seringa de 5 mL (D).

A confecção dos enxertos e do *patch* foi realizada de acordo com os princípios básicos de anastomoses vasculares descrito por Carrel^9^, Guthrie^10^ e Rutherford^11^ utilizando-se chuleio com fios de polipropileno cardiovascular 5.0 (Figura 2).

**Figura 2:** Confecção de sutura vascular: fio utilizado nas suturas (A); simulação de arteriorrafia (B).

Diversos tipos de anastomoses foram testados utilizando-se o mesmo modelo experimental: anastomoses término-terminal, látero-lateral, término-lateral, como evidenciado na Figura 3. Também foram simulados *patch*, inserção *shunt* vascular, esses simulando um cenário de endarterectomia carotídea, e tromboembolectomia utilizando-se cateter de Fogarty® (Figura 4).

**Figura 3:** Simulações realizadas: enxerto com anastomoses término-terminais (A); *patch* (B); anastomose látero-lateral (C); e enxerto com anastomoses término-laterais (D).

**Figura 4:** Cenário de endarterectomia carotídea: implante de *shunt* de carótida (A) e realização *patch* (C). Simulação de tromboembolectomia: tração do cateter insuflado em posição intraluminal (B); fragmento de gelatina removido (simulando o trombo) e o cateter de Fogarty® utilizado (D).

A perviedade das anastomoses foi comprovada pela redistribuição da gelatina intraluminal após a remoção das pinças de Bulldog; sendo também avaliado o extravasamento de gelatina entre os pontos nas suturas vasculares (anastomoses, suturas laterais e *patch*).

## RESULTADOS

Após o término das suturas, foram observadas boa coaptação das bordas e perviedade de todas as anastomoses, demonstradas pela redistribuição da gelatina intraluminal após a liberação dos clampes.

Foi possível a realização das seguintes técnicas cirúrgicas vasculares: sutura lateral (arteriorrafia), anastomoses término-terminal, látero-lateral e término-lateral, simulando confecção de enxertos e *patches*, tromboembolectomia e implante de *shunt* vascular temporário.

Não foi observado extravasamento significativo da gelatina, contudo, por tratar-se de substância coloide, com dispersão gradual, observou-se extravasamento discreto entre os pontos e, eventualmente, pelo próprio orifício gerado pela transfixação do balão pela agulha.

O custo total do modelo produzido foi de R$ 88,99, conforme demonstrado na Tabela 1. Não estão incluídos nesse orçamento os gastos com materiais permanentes, como os instrumentais cirúrgicos.

**Tabela 1:** Custos relacionados ao modelo de treinamento.

**Descrição**	**Valor unitário**
Saco de balão cilíndrico com 50 unidades	4,49
Tábua de carne de plástico	5,59
Fita adesiva dupla face	10,50
Caixa de fio de sutura (polipropileno cardiovascular 5.0) com 24 unidades	68,41
Total	88,99

## DISCUSSÃO

A utilização de simuladores para o aperfeiçoamento de habilidades cirúrgicas já foi validada há anos; contudo, os modelos mais utilizados são os cadáveres e os animais de experimentação^4^. Desde o advento da lei Arouca (Lei nº 11.794/08)^12^, um dos objetivos da comunidade científica tem sido o de diversificar os modelos de simulação, reduzindo a utilização de animais e permitindo treinamento repetitivo de técnicas cirúrgicas, formando cirurgiões mais preparados e reduzindo os riscos aos pacientes.

Diversos modelos de treinamento em suturas vasculares já foram descritos na literatura. Utilizando microscópio cirúrgico, tanto Webster & Ely^13^ quanto Lima et al.^14^ descreveram o treinamento para suturas e anastomoses término-terminais em placas e tubos de silicone de 0,6 e 1 mm. Esses autores utilizaram fios de nylon com espessura variando de 8-0 a 10-0 e, após o treinamento inicial em silicone, utilizaram ratos vivos e membros de aves abatidas para o refinamento técnico^13^^,^ 14.

Entre os modelos com materiais orgânicos, está descrita a utilização de traqueia e esôfago de frangos, simulando a consistência de artérias e veias respectivamente^7^, vasos da língua bovina^4^ e membros humanos recém-amputados^5^. Entretanto, a utilização desses materiais sempre acarreta cuidados devido ao risco de contaminação biológica. Entre esses cuidados, estão: a utilização de materiais de proteção como luvas, máscaras e óculos, necessitando ainda de descarte apropriado do material orgânico, além dos cuidados com a higiene do laboratório.

Um modelo descrito por Grahem et al.^1^ utilizou vegetais com estrutura tubular, como vagem e feijão verde, descrevendo um modelo de baixo custo para treinamento de anastomose término-terminal. A consistência e maleabilidade dos vegetais provavelmente não permitiria técnicas mais refinadas como as anastomoses término-laterais, o *patch* e outras descritas no presente estudo.

A utilização de balões de látex para o treinamento de anastomoses vasculares foi descrita por Sarmento et al.^15^, que relataram que sua maleabilidade, seu formato cilíndrico, sua espessura delgada e seu lúmen interno mostram-se semelhantes aos dos vasos sanguíneos. Nesse modelo, os balões não foram preenchidos com conteúdo intraluminal, diferente do presente estudo, no qual foi utilizada gelatina, o que permitiu que os balões permanecem túrgidos. Outra diferença em relação ao modelo anteriormente proposto é a fixação dos balões: no modelo previamente descrito, os balões são mantidos suspensos por parafusos sobre uma placa de suporte, sem manter contato com a sua superfície.

Em uma fase piloto deste estudo os balões foram presos às placas de suporte apenas por suas extremidades, por meio de grampos metálicos; no entanto, foi verificado que, ao realizar a secção transversal completa do balão, precedendo a anastomose término-terminal, o balão apresentava-se “solto” sobre a placa, mantendo-se fixo apenas pelos grampos. Em uma situação real, os vasos permanecem aderidos aos tecidos adjacentes, conferindo relativa imobilidade e facilitando as suturas, por isso optou-se pela substituição dos grampos metálicos pela fita adesiva dupla-face ao longo de todo o comprimento do balão.

Embora sem aumento considerável de custos, a aderência proporcionada pela fita e a manutenção da turgência do balão pela gelatina tornaram o modelo mais fidedigno. Além disso, essas modificações permitiram não apenas o treinamento de suturas e anastomoses, como também o acréscimo de procedimentos mais complexos, como a tromboembolectomia e a colocação do *shunt* vascular.

Além do baixo custo, outras vantagens observadas foram que, ao longo do comprimento de um único balão (28 cm), podem ser treinadas várias anastomoses/suturas vasculares e, como nenhum dos materiais utilizados é perecível, eles podem ser acondicionados por longos períodos.

Na maioria dos modelos de baixo custo de anastomose vascular, uma das limitações é avaliar a qualidade da distância entre os pontos, visto que, por tratarem-se de modelos sintéticos, há ausência de fatores de coagulação que reduzem o extravasamento entre os pontos^1^^,^ 15. Apesar de mantidos os intervalos de aproximadamente 1 mm entre os pontos, foi observado extravasamento discreto da gelatina, o que já era esperado devido às características físicas do material. Em contrapartida, a expansividade peculiar da gelatina também tornou possível atestar a perviedade das anastomoses, visto que, uma vez retirados os clampes, a gelatina se desloca de modo a preencher o espaço; outros modelos dependiam da injeção intraluminal de líquidos para atestar a perviedade das anastomoses^1^^,^ 5 ^,^ 7 ^,^ 15.

Na fase piloto, foi testado o modelo utilizando-se fio de polipropileno 6-0. Embora a realização de todos os procedimentos seja factível, a tração do fio precisa ser mais cuidadosa, pois, devido ao atrito entre o fio e o látex do balão, o fio mais fino quebra com maior facilidade ao ser tracionado. Também foram testados cateteres de Fogarty números 3 e 4. Embora seja possível a remoção da gelatina intraluminal com ambos, foi verificado, na fase inicial do treinamento, que o balão do cateter número 3 pode romper com mais facilidade durante a manipulação.

Consideramos interessante a criação de um roteiro de treinamento que correlacione as várias técnicas simuladas, com o intuito de representar o encontrado em situações reais. Por exemplo: para a “construção” de uma bifurcação arterial, uma anastomose término-lateral precisa ser realizada, permitindo a realização, em seguida e nessa mesma estrutura, de uma “arteriotomia” transversal simulando uma embolectomia no nível da bifurcação femoral ou uma “arteriotomia” longitudinal como no bulbo carotídeo, demonstrando-se a colocação do *shunt* e a síntese com *patch*.

Este modelo foi inicialmente desenvolvido para um curso de cirurgia do trauma desenvolvido por um dos autores e ministrado na nossa instituição. Naquela ocasião, residentes de cirurgia geral simularam arteriorrafias e anastomoses término-terminais. Posteriormente, o modelo foi aperfeiçoado para os outros procedimentos descritos. Nessa fase, as simulações de procedimentos foram realizadas por um dos autores, cirurgião vascular, auxiliado por alunos de graduação estagiários do grupo de pesquisa experimental da nossa faculdade de medicina. Nosso objetivo, ao publicar este artigo, era compartilhar as instruções para montagem e aplicação do modelo; a continuação da pesquisa incluirá a avaliação o grau de satisfação e o impacto sobre o treinamento dos usuários.

Os procedimentos neste modelo foram realizados com e sem o auxílio de magnificação óptica por lupa de microcirurgia, e acreditamos que esse modelo também possa ser utilizado com a finalidade de refinamento técnico para o treinamento com magnificação óptica.
